# Impact of clinical pharmacist interventions in psychiatric hospitals on clinical and medication-related outcomes: a systematic review and meta-analysis

**DOI:** 10.3389/fphar.2026.1945082

**Published:** 2026-10-08

**Authors:** Matej Stuhec, Kaja Balasko, Anteja Gorjan Gazdag, Zala Cuk, Igor Locatelli

**Affiliations:** 1 Department of Pharmacology, Faculty of Medicine Maribor, University of Maribor, Maribor, Slovenia; 2 Department of Clinical Pharmacy, Ormoz Psychiatric Hospital, Ormoz, Slovenia; 3 Faculty of Pharmacy, University of Ljubljana, Ljubljana, Slovenia

**Keywords:** clinical pharmacy, drug-related problems, mental disorders, meta-analysis, psychiatric hospitals

## Abstract

**Introduction:**

Clinical pharmacist interventions have been shown to improve outcomes and reduce drug-related problems (DRPs) in patients with mental disorders. However, no previous meta-analysis has comprehensively evaluated their effectiveness in reducing DRPs and optimizing pharmacotherapy in psychiatric inpatient settings.

**Aim:**

To evaluate the effectiveness of clinical pharmacist interventions in psychiatric inpatient settings on DRPs and clinical outcomes among patients with mental disorders.

**Methods:**

This systematic review and meta-analysis was conducted according to the PRISMA 2020 statement and prospectively registered in PROSPERO (CRD420261309290). PubMed, Scopus, CENTRAL, and CINAHL were searched from inception to March 2026. Eligible studies included adult psychiatric inpatients receiving clinical pharmacist interventions, including different pharmaceutical services, such as medication review, medication reconciliation, and ward round participation. A meta-analysis was performed in R using a random-effects model. Heterogeneity was calculated using I^2^. Random-effects meta-analyses were performed for DRPs, potentially inappropriate medications (PIMs), and the number of prescribed medications. Risk of bias was assessed using RoB two and ROBINS-I.

**Results:**

Eighteen studies met the inclusion criteria, including one randomized controlled trial and 17 non-randomized interventional studies. Clinical pharmacist interventions significantly reduced the number of prescribed medications (mean difference −1.0 medications per patient, (95% CI −1.5 to −0.5) and were associated with a pooled reduction in DRPs of 70.7% (95% CI 46.5–87.0), as well as reductions in PIMs. Evidence for patient-centred clinical outcomes, including symptom improvement, quality of life, and healthcare utilization, remained limited. Substantial heterogeneity was observed across studies, reflecting differences in intervention types, patient populations, and outcome measures. Most non-randomized studies were judged to have a serious risk of bias because of confounding and uncontrolled study designs.

**Conclusion:**

Clinical pharmacist interventions in psychiatric inpatient settings improve medication-related outcomes by reducing DRPs, PIMs, and the number of prescribed medications. These findings support the integration of clinical pharmacists into multidisciplinary psychiatric teams. Further adequately powered multicentre randomized controlled trials are needed to determine whether these improvements translate into better long-term patient-centred clinical outcomes.

## 1 Introduction

Mental disorders are among the leading contributors to the global burden of disease and are frequently associated with somatic comorbidities, resulting in reduced quality of life, increased healthcare costs, and premature mortality (Wittchen et al., 2011; Malhi and Mann, 2018). Psychiatric inpatients often receive multiple psychotropic medications together with treatments for somatic comorbidities, including cardiovascular disease, diabetes, respiratory disorders, endocrine diseases, infectious diseases, and other chronic conditions (Stuhec et al., 2025; Bitter et al., 2017; Steffen et al., 2020). Consequently, psychiatric inpatients are particularly vulnerable to polypharmacy, drug–drug interactions (DDIs), adverse drug events (ADEs), prescribing omissions, medication discrepancies, and other DRPs (Stuhec et al., 2025; Snyder et al., 2014). Polypharmacy, defined as the use of five or more medications, is highly prevalent among psychiatric inpatients and has been associated with poorer clinical outcomes, increased healthcare costs, and higher mortality (Stuhec, 2022; Maher et al., 2014; Leelakanok et al., 2003; Stuhec and Zorjan, 2022). The most common DRPs include DDIs, potentially inappropriate medications (PIMs), prescribing errors, and medication discrepancies (Stuhec, 2022; Maher et al., 2014; Leelakanok et al., 2003). PIMs are medications for which the potential risks may outweigh the expected clinical benefits, particularly when safer or more effective alternatives are available. In older adults with mental disorders, PIMs are highly prevalent, with psychotropic medications accounting for most inappropriate prescriptions, particularly benzodiazepines and antipsychotics (Lau et al., 2005; Albert et al., 2010). The high prevalence of drug-related problems (DRPs) underscores the importance of structured medication review, continuous pharmacotherapy monitoring, and interdisciplinary collaboration to optimize pharmacotherapy in psychiatric patients.

The role of clinical pharmacists has evolved from medication dispensing to active participation in multidisciplinary patient care. Clinical pharmacists contribute to optimizing pharmacotherapy for both mental disorders and somatic comorbidities, the latter being a major contributor to the excess morbidity and mortality observed in people with severe mental illness (Stuhec et al., 2025; Stuhec, 2022; Stuhec and Zorjan, 2022). Core clinical pharmacist activities include medication review, medication reconciliation, and participation in multidisciplinary ward rounds (Stuhec et al., 2025; Stuhec, 2022; Stuhec and Zorjan, 2022). Several studies have demonstrated that pharmacist-led medication reviews identify clinically significant DRPs and optimize both psychotropic and non-psychiatric pharmacotherapy (Stuhec et al., 2025; Stuhec and Zorjan, 2022). Participation in multidisciplinary ward rounds has been associated with high physician acceptance of pharmacist recommendations and improved prescribing quality (Stuhec et al., 2024; Stuhec and Tement, 2021). Likewise, pharmacist-led medication reconciliation has consistently reduced medication discrepancies during hospital admission and discharge (Oliveira et al., 2022; Vargas et al., 2023; O'Connell and VandenBerg, 2021).

However, despite these findings, no meta-analysis has comprehensively evaluated the effectiveness of clinical pharmacist interventions targeting DRPs in psychiatric inpatient settings (Werremeyer et al., 2020; Wien et al., 2024; Pimenta et al., 2026). The only previous systematic review, which included studies published between 2014 and 2019, reported that integration of psychiatric pharmacists into multidisciplinary healthcare teams was associated with improved patient-level outcomes. However, the review included heterogeneous patient populations, neurological as well as psychiatric disorders, diverse clinical settings, and various models of pharmacist intervention, limiting the applicability of its findings to psychiatric inpatient care (Werremeyer et al., 2020). Another systematic review published in 2024 focused on the prevalence of DRPs and interventions to reduce DRPs in psychiatric inpatient settings. The review included 88 studies comprising more than 95,000 psychiatric inpatients and reported a DRP prevalence ranging from 0.32 to 9.48 per patient. Prescribing errors and DDIs were the most frequently identified DRPs. Among the 13 interventional studies, clinical pharmacists were involved in seven and were shown to contribute to the identification and resolution of DRPs through interdisciplinary collaboration. However, the review did not include a meta-analysis because of the substantial heterogeneity of the included studies and synthesized evidence published between 1999 and 2023 (Wien et al., 2024). More recently, a systematic review and meta-analysis of seven randomized controlled trials in patients with schizophrenia and bipolar affective disorder also demonstrated beneficial effects of pharmacist interventions. Nevertheless, the review focused primarily on educational interventions, medication adherence, and quality of life, rather than on DRPs, PIMs, or optimization of pharmacotherapy in psychiatric inpatient settings (Pimenta et al., 2026).

Therefore, the primary aim of this systematic review and meta-analysis was to evaluate the effectiveness of clinical pharmacist interventions in psychiatric inpatient settings on DRPs and clinical outcomes and optimization of pharmacotherapy among patients with mental disorders.

## 2 Methods

### 2.1 Protocol registration

This systematic review and meta-analysis was conducted in accordance with the Preferred Reporting Items for Systematic Reviews and Meta-Analyses (PRISMA) 2020 Statement (Page et al., 2021). The review protocol was prospectively registered in the International Prospective Register of Systematic Reviews (PROSPERO) under registration number CRD420261309290 (version 2.0; published 12 June 2026). No deviations from the registered protocol were made.

### 2.2 Eligibility criteria

The eligibility criteria for this systematic review were defined according to the PICOS framework. The population included adults (≥18 years) hospitalised in psychiatric inpatient settings with any mental disorder diagnosed according to recognised diagnostic criteria. Eligible studies were conducted in specialised psychiatric hospitals or psychiatric wards within general hospitals. Studies conducted exclusively in outpatient, community, primary care, non-psychiatric inpatient, long-term care, or rehabilitation settings were excluded.

The intervention consisted of clinical pharmacist-led services delivered within psychiatric inpatient settings. Eligible interventions included participation in multidisciplinary ward rounds, structured medication reviews, identification and resolution of medication-related problems, optimisation of psychotropic therapy, and collaboration with psychiatrists and other healthcare professionals as part of routine inpatient care. Studies evaluating interventions limited to medication dispensing or supply, stock management, formulary administration, or administrative or logistical pharmacy services without direct clinical involvement in patient care were excluded. Studies evaluating educational interventions alone (e.g., one-off staff training or guideline dissemination) without patient-level clinical pharmacist activities were also excluded. In addition, studies in which the pharmacist intervention was not clearly described, could not be distinguished from other multidisciplinary interventions, or was not primarily delivered by a clinical pharmacist were excluded. Studies comparing two different clinical pharmacist interventions (e.g., medication review versus medication reconciliation) without a non-pharmacist comparator were also excluded.

The comparator was usual psychiatric inpatient care without structured involvement of a clinical pharmacist in direct clinical activities. Studies using concurrent control groups, historical controls, or pre–post designs were eligible. Studies without a comparator or pre–post assessment of outcomes were excluded.

The outcomes of interest included medication-related, clinical, healthcare utilisation, medication process, patient safety, and patient-reported outcomes. The primary outcomes were medication-related outcomes, including DRPs, PIMs, number of prescribed medications, and adverse drug events (ADEs), as well as patient-level clinical outcomes, including symptom improvement. Secondary outcomes included healthcare utilisation measures (e.g., length of hospital stay and hospital readmissions), medication process measures (e.g., pharmacist recommendations and acceptance rates), patient safety indicators (e.g., medication incidents), and patient-reported outcomes (e.g., quality of life and patient satisfaction), where reported.

Eligible study designs included randomised controlled trials (RCTs), non-randomised controlled trials, controlled before–after studies, cohort studies, and pre–post interventional studies. Case reports, case series without a comparator or pre–post evaluation, cross-sectional studies without assessment of intervention effects, qualitative studies, narrative reviews, systematic reviews, editorials, letters, conference abstracts without full text, and animal studies were excluded.

### 2.3 Information sources and search strategy

The literature search was conducted in the following electronic databases: CENTRAL (Cochrane Central Register of Controlled Trials), CINAHL (Cumulative Index to Nursing and Allied Health Literature), PubMed, and Scopus. Both published and unpublished studies were considered. No language or publication date restrictions were applied. The final literature search was conducted on 19 March 2026. Additional studies were identified by screening the reference lists of eligible articles (backward citation searching), searching relevant trial or study registries, and, where necessary, contacting study authors for additional information.

The search strategy was based on three main concepts: clinical pharmacist, mental disorders, and hospitals. Appropriate controlled vocabulary (e.g., MeSH terms where applicable) and free-text terms were combined using Boolean operators (AND/OR) and adapted for each database. The complete electronic search strategy for all databases, including database-specific adaptations, is provided in Supplementary File 2 – Complete Search Strategies and Additional Data.

### 2.4 Study selection

Two reviewers (K.B. and A.G.G.) independently screened the titles and abstracts of all retrieved records for eligibility. Full-text articles of potentially eligible studies were independently assessed by the same two reviewers. Any disagreements regarding study eligibility were resolved through discussion between I.L. and M.S. All retrieved records were imported into Microsoft Excel® for duplicate removal before the screening process. The study selection process was documented using the PRISMA 2020 flow diagram.

### 2.5 Data extraction

Data were independently extracted by two reviewers (K.B. and A.G.G.) using a standardised data extraction form developed in Microsoft Excel®. Extracted data included the first author, year of publication, country, study design, sample size, participant characteristics, psychiatric diagnoses, characteristics of the clinical pharmacist intervention (including the type of medication review), comparator, outcome measures (e.g., DRPs, PIMs, ADEs, number of medications, healthcare utilisation, quality of life, and other reported outcomes), and the main study findings. Disagreements were resolved through discussion, with I.L. and M.S. consulted when necessary. Detailed characteristics of the included studies are provided in Table 1.

**TABLE 1:** Characteristics of the included studies.

First author, year	Country	Study design	Sample size (n)	Type of intervention	DRPs	PIMs	No. of medications	ADEs	QoL	Cost
Engel-Dettmers, 2008	Netherlands	Prospective before–after study	n = 188	Medication review (type 1)	NO	NO	NO	NO	NO	NO
Snyder, 2014	USA	Retrospective before–after study	n = 29	Medication review (type 2b)	NO	YES	NO	YES	NO	NO
Hashimoto, 2016	Japan	Prospective before–after study	n = 52	Medication review (type 2b)	NO	NO	YES	NO	NO	YES
Ilickovic, 2016	Montenegro	Prospective controlled before–after study	n = 49	Medication review (type 2b)	YES	NO	YES	NO	NO	NO
Graveland, 2016	Netherlands	Prospective before–after study	n = 70	Medication review (type 3)	NO	NO	YES	NO	NO	YES
Shin, 2020	Japan	Retrospective before–after study	n = 69	Medication review (type 2b)	NO	NO	YES	NO	NO	YES
Margulis, 2021	USA	Retrospective controlled before–after study	n = 111	Medication review (type 3)	NO	NO	NO	NO	NO	NO
Sriboonruang, 2024	Thailand	Randomized controlled trial study	n = 150	Medication review (type 3)	YES	NO	NO	YES	YES	NO
Stuhec, 2025	Slovenia	Retrospective before–after study	n = 100	Medication review (type 3)	YES (only DDIs)	YES	YES	NO	NO	NO
Hahn, 2018	Germany	Prospective before–after study	n = 48	Participation in multidisciplinary ward rounds	YES (only DDIs)	YES	YES	NO	NO	NO
Stuhec, 2021	Slovenia	Retrospective before–after study	n = 224	Participation in multidisciplinary ward rounds	YES	NO	NO	NO	NO	NO
Stuhec, 2024	Slovenia	Retrospective before–after study	n = 186	Participation in multidisciplinary ward rounds	YES	NO	NO	NO	NO	NO
Lizer, 2007	USA	Prospective before–after study	n = 54	Medication reconciliation	NO	NO	NO	NO	NO	NO
O'Connell, 2021	USA	Retrospective controlled before–after study	n = 198	Medication reconciliation	YES (medication errors)	NO	NO	NO	NO	NO
Oliveira, 2022	Portugal	Prospective before–after study	n = 148	Medication reconciliation	NO	NO	NO	NO	NO	NO
Vargas, 2023	USA	Retrospective before–after study	n = 72	Medication reconciliation	YES (medication errors)	NO	NO	NO	NO	NO
Saklad, 1984	USA	Retrospective controlled before–after study	n = 61	Clinical pharmacy intervention	NO	NO	YES	NO	NO	NO
Hahn, 2013	Germany	Retrospective controlled before–after study	n = 200	DDIs review	YES (only DDIs)	NO	NO	NO	NO	NO

Abbreviations: ADEs, adverse drug events; DDIs, drug–drug interactions; DRPs, drug-related problems; PIMs, potentially inappropriate medications; QoL, quality of life.

### 2.6 Data synthesis and statistical analysis

Statistical analyses were performed by K.B. and A.G.G. and independently checked by I.L. and M.S. Statistical analyses were conducted using the R statistical environment (version 4.6.1; R Foundation for Statistical Computing, Vienna, Austria) with the meta package. Meta-analyses were performed when studies were considered sufficiently homogeneous with respect to the study population, intervention, comparator, and outcome measures. Studies were included in the quantitative synthesis only when the reported data were sufficiently comparable and provided the information required to calculate the corresponding effect estimate; otherwise, they were retained in the qualitative synthesis. Studies without a comparator or pre–post assessment of outcomes were excluded; acceptance of pharmacist recommendations alone was considered a medication-process measure and not a pre–post assessment of the corresponding clinical or medication-related outcome. Because clinical and methodological heterogeneity across the included studies was anticipated, random-effects models were used throughout. Effect estimates were pooled using the inverse-variance method, and between-study variance (τ^2^) was estimated using restricted maximum likelihood (REML). Confidence intervals for τ^2^ and τ were calculated using the Q-profile method. Hartung–Knapp adjustments were applied to calculate confidence intervals for the pooled random-effects estimates. Statistical heterogeneity was assessed using Cochran’s Q test and the I^2^ statistic, with I^2^ calculated based on Q.

For continuous outcomes, specifically the number of prescribed medications, mean differences (MDs) between after-intervention and before-intervention values were calculated and reported with 95% confidence intervals (CIs). For proportional outcomes, including reductions in DRPs and PIMs, study-specific proportions were transformed using the logit transformation and pooled using an inverse-variance random-effects model. Clopper–Pearson exact binomial CIs were calculated for the proportions reported by individual studies. Pooled estimates were back-transformed to the original scale and expressed as events per 100 participants. Where applicable, 95% prediction intervals were calculated using the t-distribution to indicate the expected range of effects in a future comparable study.

Meta-analyses were conducted for DRPs, PIMs, and the number of prescribed medications. Other outcomes, including clinical outcomes, healthcare utilisation, medication process measures, patient safety indicators, and patient-reported outcomes, were synthesised narratively because of an insufficient number of studies, methodological heterogeneity, or insufficient quantitative data. A leave-one-out sensitivity analysis was performed to evaluate the influence of individual studies on the pooled estimates and to assess the robustness of the meta-analysis results.

### 2.7 Risk of bias assessment

The risk of bias of the included studies was independently assessed by two reviewers (K.B. and A.G.G.), with disagreements resolved through discussion and, where necessary, consultation with I.L. and M.S. The Cochrane Risk of Bias 2 (RoB 2) tool was used to assess randomised controlled trials, whereas the Risk Of Bias In Non-randomized Studies of Interventions (ROBINS-I) tool was used for non-randomised studies (Sterne et al., 2019; Sterne et al., 2016). Where required, additional information was sought from the study authors to clarify methodological details.

Publication bias was planned to be assessed through visual inspection of funnel plots and statistical tests for small-study effects where appropriate. However, publication bias was not formally assessed because each meta-analysis included fewer than ten studies, making these methods unreliable.

## 3 Results

### 3.1 Study selection

A total of 1,604 records were identified through the systematic literature search. Before screening, 711 records were removed, because they were identified as duplicates. The remaining 893 records were screened based on titles and abstracts. Following the screening process, 66 full-text reports were sought for retrieval, of which 61 were assessed for eligibility. After full-text assessment, 18 studies met the predefined inclusion criteria and were included in the systematic review. The study selection process and reasons for exclusion are presented in the PRISMA 2020 flow diagram (Figure 1). The PRISMA 2020 Checklist has been attached (Supplementary File 3).

**FIGURE 1:**
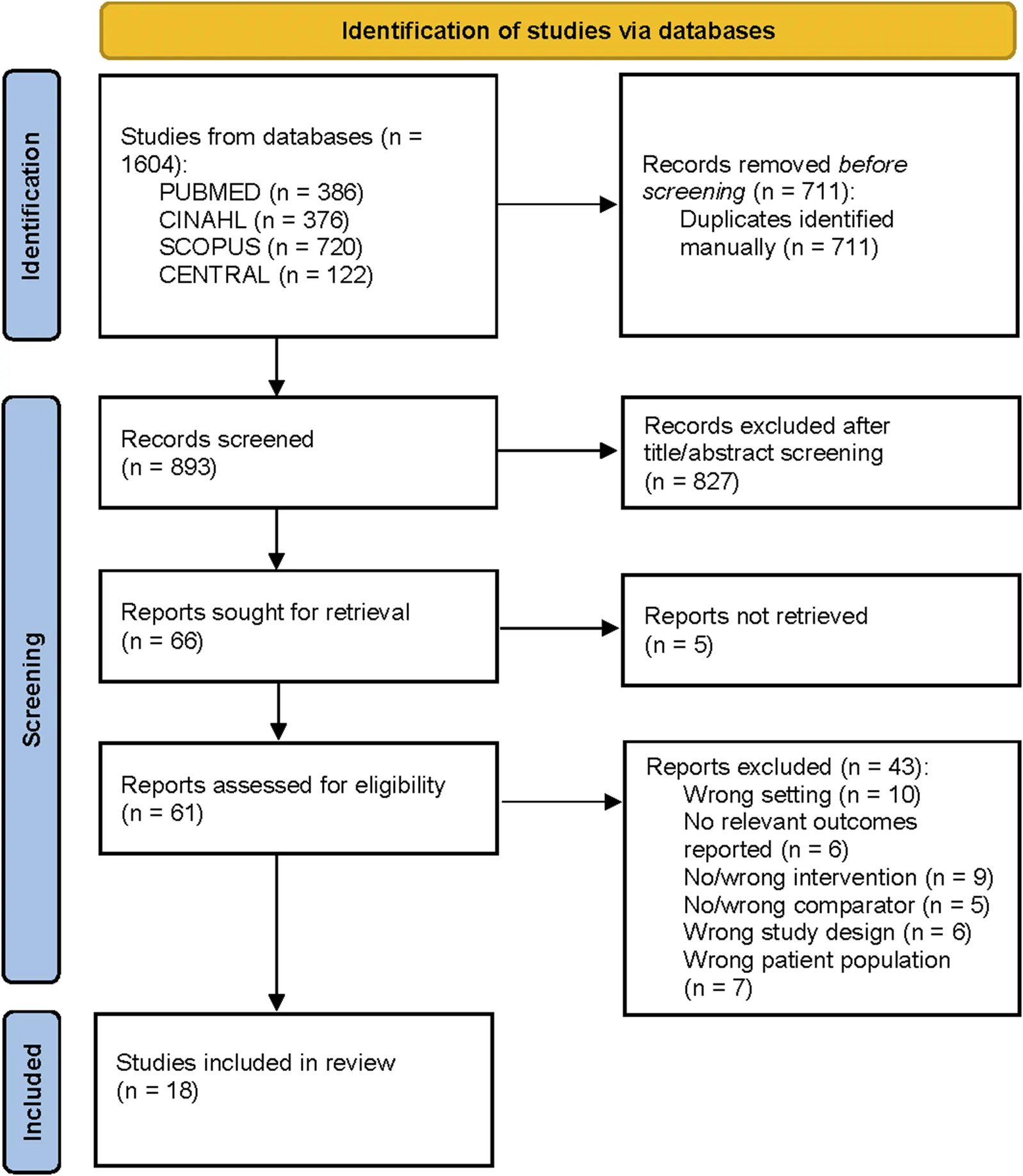
The PRISMA flow diagram with a summary of the study selection process, including the reasons for exclusion at each stage.

### 3.2 Characteristics of included studies

A total of 18 studies published between 1984 and 2025 were included in this systematic review. The studies were conducted in eight countries, including the United States, Slovenia, the Netherlands, Germany, Japan, Portugal, Montenegro, and Thailand. All included studies were conducted in psychiatric inpatient settings. Detailed characteristics of the included studies are presented in Table 1 and Supplementary File 1.

The methodological designs of the included studies were heterogeneous. Among the 18 included studies, 17 were before–after studies and one was a randomized controlled trial (RCT) (Stuhec et al., 2025; Snyder et al., 2014; Stuhec et al., 2024; Stuhec and Tement, 2021; Oliveira et al., 2022; Vargas et al., 2023; O'Connell and VandenBerg, 2021; Saklad et al., 1984; Engel-Dettmers et al., 2008; Lizer and Brackbill, 2007; Hahn et al., 2013; Hashimoto and Tensho, 2016; Ilickovic et al., 2016; Graveland et al., 2016; Hahn et al., 2018; Shin et al., 2020; Margulis et al., 2021; Sriboonruang et al., 2024). Seven before–after studies were prospective and ten retrospective. Sample sizes ranged from 29 to 224 participants.

The interventions primarily involved clinical pharmacist-led pharmaceutical care, including medication reviews (n = 9), participation in multidisciplinary ward rounds (n = 3), medication reconciliation (n = 4), and other collaborative pharmaceutical interventions (n = 2). Medication reviews ranged from Type 1 to Type 3 according to the Pharmaceutical Care Network Europe (PCNE) classification, depending on the complexity of the intervention. Most studies evaluated interventions covering all prescribed medications, whereas several were limited to specific medication groups, such as benzodiazepines or antiretroviral therapy, indicating that clinical pharmacists did not intervene across the entire pharmacotherapy regimen in every study.

The included studies investigated patients with a range of psychiatric disorders, most commonly schizophrenia and other severe mental illnesses, although several studies included mixed psychiatric inpatient populations. Clinical pharmacist interventions aimed to optimize pharmacotherapy through identifying and resolving DRPs, improving medication appropriateness, reducing polypharmacy, supporting deprescribing, and promoting adherence to evidence-based treatment guidelines.

Primary outcomes were the most frequently evaluated and included medication-related outcomes, such as the number of medications, DRPs, ADEs, and PIMs, as well as patient-level clinical outcomes, including symptom improvement. Secondary outcomes were reported less frequently and included healthcare utilisation outcomes (e.g., hospital length of stay and readmissions), medication process outcomes (e.g., acceptance of pharmacist recommendations, medication adherence, and adherence to clinical treatment guidelines), patient safety outcomes (e.g., medication incidents and falls), and patient-reported outcomes (e.g., quality of life). Overall, most studies demonstrated positive effects of clinical pharmacist interventions, particularly in reducing DRPs, optimizing pharmacotherapy, decreasing inappropriate prescribing, and improving adherence to treatment recommendations. However, the reported outcomes varied considerably across studies because of differences in study design, intervention type, outcome measures, and follow-up duration. Outcomes that were not sufficiently comparable for quantitative synthesis were synthesized narratively.

### 3.3 Risk of bias assessment

The methodological quality of the included studies was assessed using the ROBINS-I tool for non-randomized studies. The single randomized controlled trial (Sriboonruang et al., 2024) was assessed separately using the Revised Cochrane Risk of Bias tool (RoB 2) (Sriboonruang et al., 2024). This study was judged to have some concerns overall. Low risk of bias was identified for the randomization process, missing outcome data, and selection of the reported result, whereas some concerns were identified for deviations from intended interventions and measurement of outcomes because blinding procedures were not clearly reported. The detailed ROBINS-I assessment of the non-randomized studies is presented in Table 2.

**TABLE 2:** ROBINS-I risk of bias assessment by domain.

Study	D1	D2	D3	D4	D5	D6	D7
Engel-Dettmers, 2008	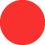	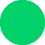	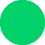	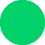	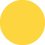	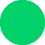	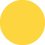
Graveland, 2016	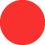	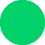	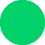	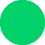	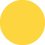	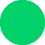	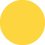
Hahn, 2013	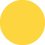	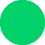	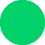	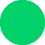	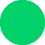	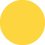	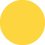
Hahn, 2018	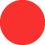	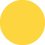	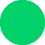	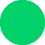	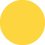	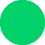	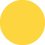
Hashimoto, 2016	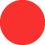	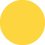	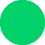	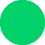	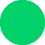	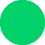	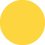
Ilickovic, 2016	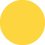	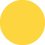	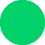	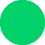	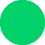	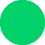	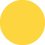
Lizer, 2007	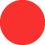	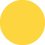	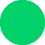	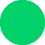	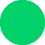	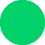	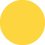
Margulis, 2021	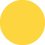	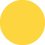	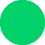	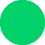	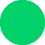	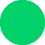	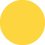
O'Connell, 2021	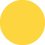	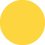	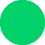	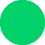	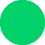	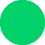	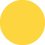
Oliveira, 2022	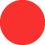	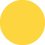	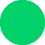	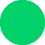	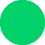	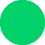	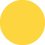
Saklad, 1984	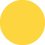	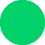	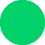	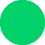	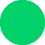	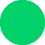	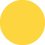
Shin, 2020	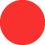	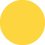	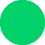	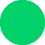	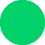	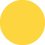	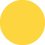
Snyder, 2014	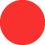	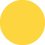	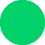	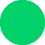	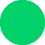	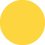	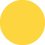
Stuhec, 2021	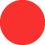	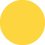	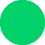	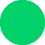	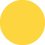	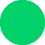	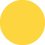
Stuhec, 2024	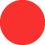	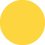	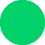	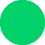	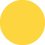	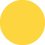	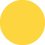
Stuhec, 2025	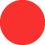	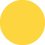	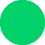	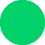	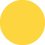	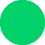	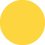
Vargas, 2023	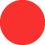	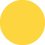	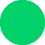	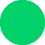	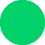	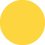	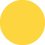

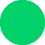
 Low risk of bias

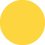
 Moderate risk of bias

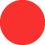
 Serious risk of bias

ROBINS-I domains

D1: Bias due to confounding

D2: Bias in selection of participants

D3: Bias in classification of interventions

D4: Bias due to deviations from intended interventions

D5: Bias due to missing data

D6: Bias in measurement of outcomes

D7: Bias in selection of the reported result

Seventeen of the eighteen included studies were assessed using the ROBINS-I tool. Overall, twelve studies were judged to have a serious risk of bias, whereas five studies were judged to have a moderate risk of bias. The most common source of bias across the non-randomized studies was bias due to confounding. Most studies employed uncontrolled before–after or retrospective observational designs without randomization or concurrent control groups. Consequently, improvements in prescribing practices, medication-related outcomes, or clinical outcomes could not be attributed solely to the clinical pharmacist intervention.

Studies that included a comparison cohort, including Saklad et al. (1984), Hahn et al. (2013), Ilickovic et al. (2016), Margulis et al. (2021), and O'Connell and VandenBerg (2021), demonstrated a lower overall risk of bias and were therefore rated as having moderate risk of bias (O'Connell and VandenBerg, 2021; Saklad et al., 1984; Hahn et al., 2013; Ilickovic et al., 2016; Margulis et al., 2021). Although these studies were not randomized, the presence of comparison groups reduced the likelihood that the observed effects resulted solely from temporal changes.

Across most studies, the ROBINS-I domains bias in classification of interventions and bias due to deviations from intended interventions were judged as low risk of bias, reflecting the clear description and consistent implementation of pharmacist-led interventions within routine clinical practice. Likewise, bias in measurement of outcomes was generally considered to present low or moderate risk because most studies relied on objective outcome measures, including medication records, DRPs, medication discrepancies, DDIs, PIMs, medication costs, hospital readmissions, and length of stay.

The domains bias in selection of participants, bias due to missing data, and bias in selection of the reported result were most frequently judged as presenting moderate risk of bias. Selection bias primarily resulted from retrospective recruitment, restrictive eligibility criteria, or exclusion of patients with incomplete data. Moderate risk due to missing data was identified in studies with incomplete follow-up or unavailable clinical information. Furthermore, none of the included non-randomized studies reported a pre-registered study protocol, making selective outcome reporting difficult to exclude.

Overall, despite generally well-described interventions and objective outcome assessment, the predominance of non-randomized uncontrolled study designs resulted in an overall serious risk of bias for the majority of included studies.

### 3.4 Meta-analysis of primary outcomes

#### 3.4.1 Number of medications

Four studies reported changes in the number of medications following clinical pharmacist intervention in the meta-analysis (Figure 2). The intervention was associated with a statistically significant reduction in the number of medications, with a pooled mean difference of −1.0 medication per patient (95% CI: −1.5 to −0.5; p = 0.0074), based on a random-effects model. No relevant between-study heterogeneity was observed (I^2^ = 0.0%, τ^2^ = 0.0155; p = 0.4682). The prediction interval ranged from −1.7 to −0.3 medications, indicating that a reduction in the number of medications would also be expected in a comparable future study.

**FIGURE 2:**
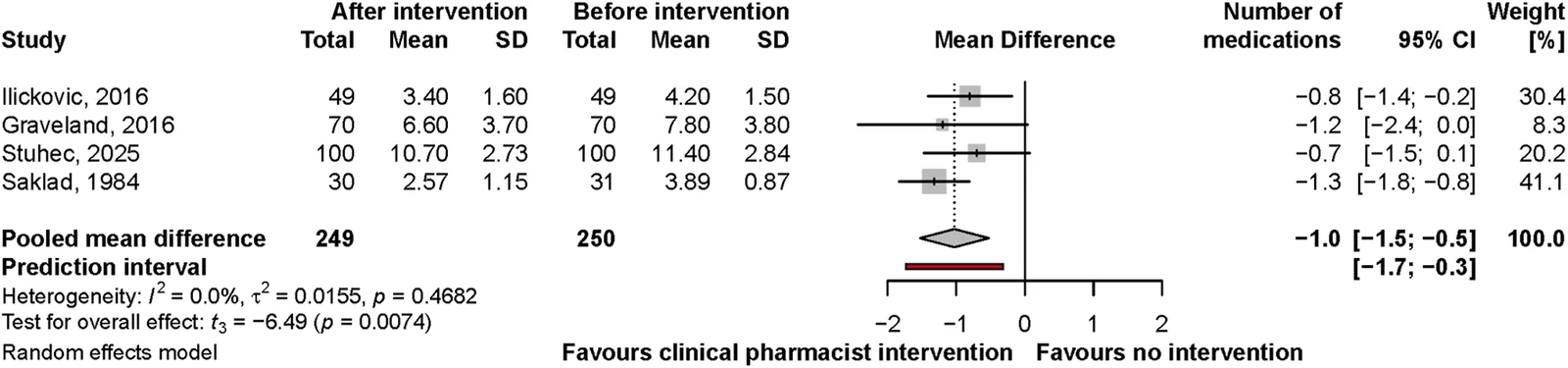
Forest plot of the mean difference in the number of medications before and after clinical pharmacist intervention.

#### 3.4.2 Drug-related problems (DRPs)

Seven studies reported the percentage of DRPs reduced following clinical pharmacist intervention in the meta-analysis (Figure 3). The pooled DRP reduction was 70.7% (95% CI: 46.5–87.0), based on a random-effects model. Between-study heterogeneity was very high (I^2^ = 96.5%, τ^2^ = 1.1601; p < 0.0001), indicating substantial variability in the reported estimates. The prediction interval was wide, ranging from 12.5% to 97.6%, suggesting that the proportion of DRPs reduced may vary considerably across different clinical settings and interventions. Individual study estimates ranged from 43.6% to 93.7%. A leave-one-out sensitivity analysis was performed to assess the influence of individual studies on the pooled estimate of DRP reduction (Supplementary File 2). Sequential omission of each study resulted in pooled DRP reductions ranging from 64.2% to 74.3%. The exclusion of Stuhec, 2021 had the greatest impact on the pooled estimate and reduced between-study heterogeneity from 96.5% to 94.3%, although heterogeneity remained very high (Supplementary File 2). Omitting any other study produced only modest changes in the pooled estimate and heterogeneity (I^2^ ranging from 96.3% to 97.1%). Overall, the findings indicate that the observed effect was robust and was not driven by any single study, despite the substantial between-study heterogeneity.

**FIGURE 3:**
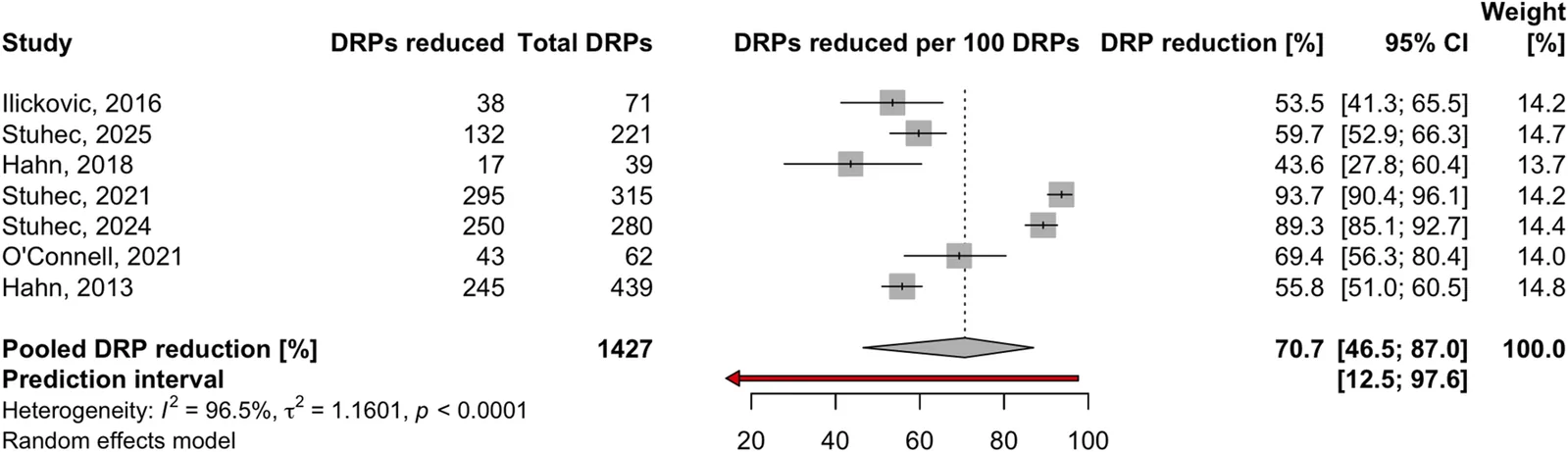
Forest plot of the percentage of DRPs reduced following clinical pharmacist intervention.

#### 3.4.3 Potentially inappropriate medications (PIMs)

Studies reported reductions in PIMs were analysed according to the criterion used to identify PIMs (Figure 4). Under the STOPP criterion, one study reported a PIM reduction of 41.1% (95% CI: 31.9–50.8); therefore, a pooled estimate and heterogeneity could not be calculated for this subgroup. For the Beers criteria, the pooled PIM reduction was 37.5% (95% CI: 0.0–100.0), with very high heterogeneity (I^2^ = 98.0%, τ^2^ = 2.1701). For the PRISCUS criteria, the pooled PIM reduction was 30.0% (95% CI: 9.7–63.1), with no observed heterogeneity (I^2^ = 0.0%, τ^2^ = 0). The wide confidence interval for the Beers subgroup and the limited number of studies within each subgroup indicate that these findings should be interpreted cautiously.

**FIGURE 4:**
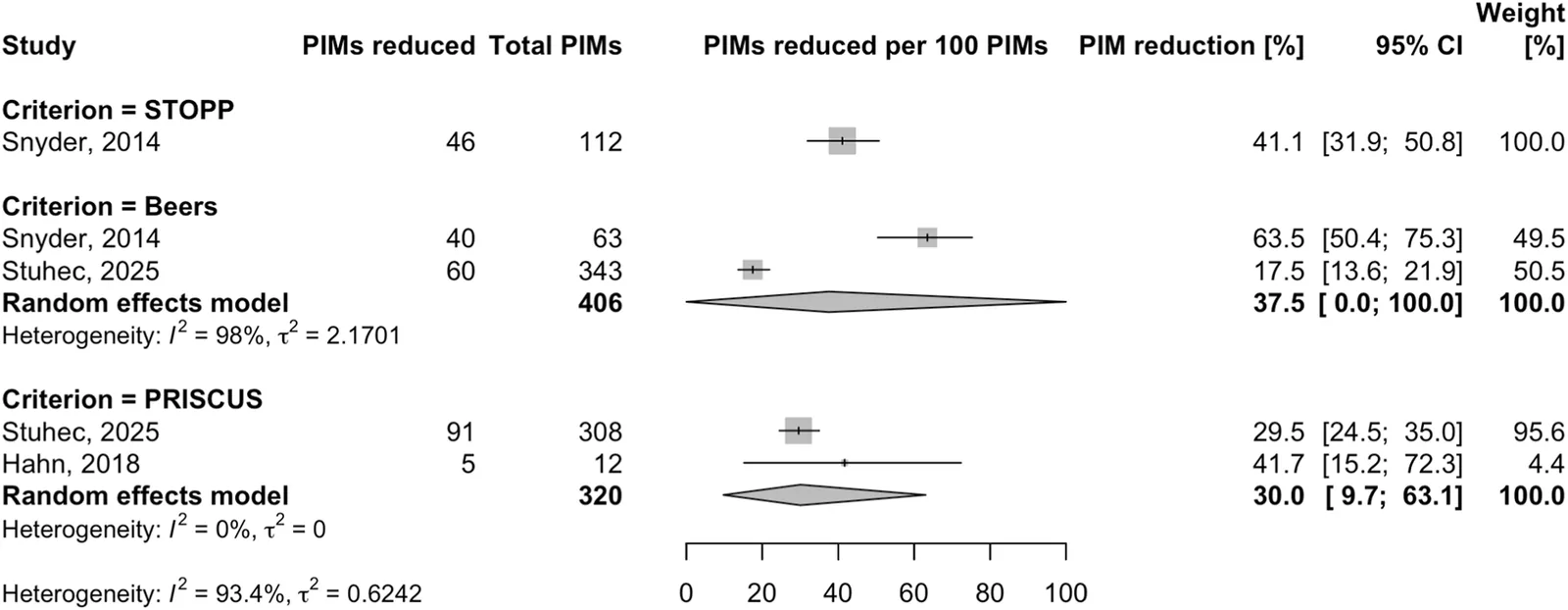
Forest plot of the percentage of PIMs reduced following clinical pharmacist intervention, stratified by the criterion used to identify PIMs.

## 4 Discussion

To our knowledge, this is the first systematic review and meta-analysis to comprehensively evaluate the impact of clinical pharmacist interventions in psychiatric inpatient settings while incorporating sensitivity analyses and structured risk of bias assessment. Unlike previous reviews, our review was restricted to psychiatric inpatient settings, quantitatively synthesized medication-related outcomes, and evaluated structured clinical pharmacist interventions, including medication review, medication reconciliation, and participation in multidisciplinary ward rounds. Overall, the findings demonstrate that clinical pharmacist involvement is consistently associated with significant reductions in DRPs, PIMs, and the overall number of prescribed medications. These findings further support the growing role of clinical pharmacists as integral members of multidisciplinary psychiatric teams (Werremeyer et al., 2020; Wien et al., 2024; Pimenta et al., 2026).

However, evidence regarding patient-centred clinical outcomes, including symptom remission, relapse prevention, quality of life, remains limited. Furthermore, the predominance of non-randomized studies substantially limits causal inference; therefore, the observed benefits should be interpreted cautiously despite the consistency of findings across the included studies.

The largest effect of clinical pharmacist interventions was observed for the reduction of DRPs. This finding is clinically plausible because psychiatric inpatients frequently receive complex psychopharmacological regimens, often involving polypharmacy, high-risk medications, DDIs, and multiple somatic comorbidities (Stuhec et al., 2025; Bitter et al., 2017; Steffen et al., 2020). These characteristics create numerous opportunities for identifying prescribing errors, optimizing treatment, and improving medication safety. Consequently, clinical pharmacists are well positioned to detect and resolve DRPs that may otherwise remain unnoticed. The magnitude of the observed reduction is comparable to findings reported in primary care and long-term care settings, where pharmacist-led medication optimization has consistently reduced inappropriate prescribing and DRPs (Stuhec and Zorjan, 2022; Stuhec, 2021). Similar findings were reported by Stuhec et al., who demonstrated significant reductions in DRPs and improvements in quality of life among older nursing home residents with mental disorders following pharmacist-led medication reviews (Stuhec et al., 2019). Likewise, Stuhec and Zorjan observed substantial improvements in psychopharmacotherapy optimization in primary care patients with mental disorders, further supporting the transferability of pharmacist-led interventions across healthcare settings (Stuhec and Zorjan, 2022). Although the present review did not evaluate clinical endpoints such as mortality, potentially inappropriate prescribing has previously been associated with an increased risk of adverse clinical outcomes, including hospitalisation and mortality, in older populations (Lau et al., 2005; Albert et al., 2010; Hamilton et al., 2009).

The substantial heterogeneity observed in the meta-analysis of DRPs was expected given the heterogeneity among the included studies. The studies differed in the types of clinical pharmacist interventions (e.g., medication review and medication reconciliation), the scope of the interventions (all prescribed medications versus selected medication groups), definitions and classifications of DRPs, patient populations, healthcare systems, and follow-up. These differences likely contributed to the variability in the magnitude of the reported effects. Nevertheless, despite the high statistical heterogeneity, all included studies consistently demonstrated a reduction in DRPs following clinical pharmacist intervention, and the leave-one-out sensitivity analysis confirmed that the overall findings were robust and not driven by a single study. Therefore, the heterogeneity appears to reflect differences in intervention implementation and study characteristics rather than directions of effect.

An important finding of this review relates to the effectiveness of different types of clinical pharmacist interventions. Medication reviews, medication reconciliation, and participation in multidisciplinary ward rounds were the most frequently evaluated services and all demonstrated favourable effects on medication optimization. Medication reviews, particularly comprehensive Type 3 PCNE reviews, represent one of the best-established clinical pharmacy services internationally and have consistently demonstrated effectiveness in identifying DRPs (Stuhec, 2021). Medication reviews were implemented in approximately half of the included studies, supporting their role as a core component of clinical pharmacy practice in psychiatric inpatient settings. Interestingly, acceptance rates of pharmacist recommendations appeared higher in psychiatric inpatient settings than those previously reported in primary care. One plausible explanation is the closer interdisciplinary collaboration between psychiatrists and clinical pharmacists within hospital wards, where face-to-face communication facilitates rapid discussion of recommendations although this hypothesis requires confirmation in future comparative studies (Stuhec et al., 2025; Stuhec and Tement, 2021).

Participation in multidisciplinary ward rounds represents another important component of psychiatric clinical pharmacy practice. Daily collaboration enables clinical pharmacists to proactively identify DRPs during treatment planning rather than retrospectively through chart review alone. Such integration into the multidisciplinary team may explain the relatively high acceptance rates of pharmacist recommendations observed across several included studies. Nevertheless, acceptance rates varied considerably between studies, suggesting that local organization, prescribing culture, and integration of pharmacists into clinical teams may influence the effectiveness of these interventions.

Medication reconciliation also emerged as an important area in which clinical pharmacists contribute to improving transition of care (Oliveira et al., 2022; Vargas et al., 2023; O'Connell and VandenBerg, 2021). Psychiatric patients are particularly vulnerable to medication discrepancies at hospital admission and discharge because medication histories are frequently incomplete, communication with patients may be challenging, and psychotropic therapy is often modified during transitions of care (Oliveira et al., 2022; Vargas et al., 2023; O'Connell and VandenBerg, 2021). Consistent with World Health Organization recommendations, pharmacist-led medication reconciliation improves the accuracy of medication histories and reduces medication discrepancies during transitions between healthcare settings (Stuhec and Batinic, 2023). The findings are further supported by Oliveira et al., who reported that pharmacist involvement identified a substantial number of clinically relevant medication discrepancies requiring clarification with psychiatrists, most commonly omitted medications (Oliveira et al., 2022). These findings highlight medication reconciliation as an important opportunity for improving continuity of care and reducing preventable medication errors in psychiatric inpatient settings.

Despite the consistent improvements in medication-related outcomes, evidence regarding patient-centred clinical outcomes remains scarce. Few studies evaluated outcomes such as psychiatric symptom improvement, relapse prevention, hospital readmissions, or quality of life, and the available evidence was insufficient for quantitative synthesis. This likely reflects both methodological limitations and the complexity of psychiatric care. Unlike medication-related outcomes, improvements in psychiatric symptoms are influenced by numerous clinical, psychological, and social factors beyond pharmacotherapy alone. Furthermore, many included studies had relatively short follow-up periods and were primarily designed to evaluate prescribing quality rather than long-term clinical effectiveness. Future studies should therefore incorporate validated patient-centred outcomes together with medication-related measures to better demonstrate the broader clinical value of pharmacist interventions.

The methodological quality of the available evidence represents an important limitation when interpreting these findings. Seventeen of the eighteen included studies were non-randomized, with most judged to have a serious risk of bias primarily because of confounding, lack of concurrent control groups, and uncontrolled before-after study designs. Nevertheless, several methodological strengths should also be acknowledged. Pharmacist interventions were generally well described, outcome assessment relied predominantly on objective measures, and reporting bias appeared relatively limited. Moreover, the consistency of findings across studies conducted in different countries and healthcare systems provides additional confidence that the observed effects are likely to represent clinical benefits.

This review has several additional limitations. First, relatively few studies met the inclusion criteria, precluding formal assessment of publication bias and limiting statistical power for several outcomes. Second, considerable heterogeneity existed regarding intervention characteristics, patient populations, outcome definitions, and follow-up duration, which may have contributed to between-study variability. Third, the included studies originated from a limited number of countries where clinical pharmacy services are relatively well established, potentially limiting the generalisability of these findings to healthcare systems with less developed psychiatric pharmacy services. Finally, this review focused exclusively on psychiatric inpatient settings, and therefore the findings should not be extrapolated to outpatient, primary care, or long-term care settings, where separate evidence has already been synthesised in previous systematic reviews.

The main strengths of this review include comprehensive literature searching, adherence to PRISMA, prospective protocol registration, structured risk of bias assessment, sensitivity analyses, and quantitative synthesis of medication-related outcomes. The findings of this review have important implications for psychiatric inpatient care. Given the consistent improvements in DRPs, hospitals should consider integrating clinical pharmacists into multidisciplinary psychiatric teams as part of routine care. Pharmacist-led medication review, medication reconciliation, and participation in multidisciplinary ward rounds appear to represent the most effective interventions for optimizing pharmacotherapy. Based on these findings, hospital administrators and healthcare policymakers should consider expanding pharmacist-led services, particularly medication review and medication reconciliation, as part of routine psychiatric inpatient care. Although evidence for patient-centred clinical outcomes remains limited, improvements in prescribing quality represent an important issue for better long-term clinical outcomes. The results observed in this review support the further integration of clinical pharmacists into multidisciplinary psychiatric teams. Emerging evidence from primary care and community mental health settings also suggests that expanding pharmacists' clinical roles, including collaborative or independent prescribing, may further improve medication optimization, patient-centred outcomes, and continuity of care (Alsaeed et al., 2025; Vaughan et al., 2026).

Despite these limitations, this review provides the strongest available synthesis of evidence regarding clinical pharmacist interventions in psychiatric inpatient settings. The consistent reductions in DRPs, inappropriate prescribing, and medication burden suggest that integrating clinical pharmacists into multidisciplinary psychiatric teams represents an effective strategy for improving medication safety and optimizing psychopharmacotherapy. Future adequately powered multicentre randomized controlled trials should evaluate whether these improvements in medication-related outcomes translate into meaningful benefits in psychiatric symptoms, healthcare utilisation, patient quality of life, and long-term clinical outcomes. Future studies should also evaluate cost-effectiveness because reductions in DRPs may translate into lower healthcare costs.

## Data Availability

All data generated or analyzed during this study are included in the article/Supplementary Material.
