# Supplementary material for: Impact of clinical pharmacist interventions in psychiatric hospitals on clinical and medication-related outcomes: a systematic review and meta-analysis
Source: Front Pharmacol. 2026 Oct 8;17:1945082. doi: 10.3389/fphar.2026.1945082 (PMC13687401; doi:10.3389/fphar.2026.1945082)
Supplement: Supplementary file 1 [file Supplementaryfile1.docx]

| **First Author, Year** | **Type of intervention** | **Outcomes** |
| --- | --- | --- |
| Engel-Dettmers, 2008 | Medication review (type 1) | Patients achieving ≥20% benzodiazepine dose reduction increased from **8.0% (baseline)** to **27% (2 weeks post-tapering)** and **31% (6 months)** (p < 0.001). Mean diazepam-equivalent dose decreased significantly from **20.0 mg** to **16.7 mg** (2 weeks) and **16.8 mg** (6 months). Patients reducing benzodiazepines were more likely to increase the use of other sedatives at **2 weeks (22.4% vs. 6.6%;** p **= 0.006)** and **6 months (36.5% vs. 17.8%;** p **= 0.007).** |
| Snyder, 2014 | Medication review (type 2b) | Mean PIMs per patient decreased significantly using STOPP (112 to 66; mean change −1.6±1.5; p < 0.0001) and Beers criteria (63 to 23; mean change −1.4±1.1; p < 0.0001), with no difference between criteria (p = 0.375). 84% of pharmacist recommendations were accepted. Falls decreased by 37.5%, referrals/transfers by 27.2% (STOPP) and 23.1% (Beers), although changes were not statistically significant. ADEs decreased significantly with both criteria; serious ADEs decreased from 11 to 6. |
| Hashimoto, 2016 | Medication review (type 2b) | Mean antipsychotic dose decreased from 982.6 to 857.6 mg (p < 0.01), and the number of antipsychotics decreased significantly (median 2.0 [1.0–6.0] to 2.0 [1.0–5.0]; p < 0.05). Antipsychotic polypharmacy decreased from 73.1% to 63.5%. Seclusion room use decreased from 44.2% to 28.8%, but not significantly (p = 0.077). |
| Ilickovic, 2016 | Medication review (type 2b) | 71 DRPs were identified in 49 patients. Pharmacists proposed 182 interventions; 91 (50%) were accepted and implemented. 38 DRPs were completely or partially resolved. The number of prescribed medications decreased significantly (4.2±1.5 to 3.4±1.6; p = 0.002), whereas antipsychotic dose remained unchanged (p = 0.686). |
| Graveland, 2016 | Medication review (type 3) | Mean number of medications decreased significantly from 7.8±3.8 to 6.6±3.7 (p < 0.001). Psychotropic medications decreased from 3.0 to 2.8 (p = 0.01), and somatic medications from 4.6 to 3.7 (p < 0.001). Of 178 proposed medication changes, 89 were implemented within 3 months and 20 additional changes during follow-up. |
| Shin, 2020 | Medication review (type 2b) | Median benzodiazepine dose decreased from 20.0 to 13.8 mg diazepam equivalents (p = 0.02), while the number of benzodiazepines decreased non-significantly (p = 0.22). Recommendation acceptance rates were high in both groups receiving the intervention, reaching 94.4% and 98.2%, with 100% acceptance of recommendations concerning high-risk drugs. Patient outcomes in the first group were 29.4% and 70.6% for “improvement” and “unchanged”, respectively. In the second group, patient outcomes were 47.7%, 50.5%, and 1.9% for “improvement”, “unchanged”, and “exacerbation”, respectively. |
| Margulis, 2021 | Medication review (type 3) | Appropriate ARV prescribing increased from 32% (Phase I - no pharmacist involvement ) to 62% (Phase II - partial pharmacist involvement; p = 0.01) and 84% (Phase III - consistent pharmacist involvement; p = 0.036). Comprehensive HIV management improved from 5% (Phase I) to 38% (Phase II; p < 0.001) and 46% (Phase III; p = 0.48). Appropriate OI prophylaxis increased from 11% to 50% and 57%. Use of single-tablet regimens increased from 30% to 49% and 57%. |
| Sriboonruang, 2024 | Medication review (type 3) | The proportion of patients with DRPs did not differ significantly between groups (p = 0.671), but resolved DRPs were significantly more frequent in the intervention group (86.4% vs. 18.8%; p < 0.001). ADRs (57.6% vs. 79.7%; p = 0.007) and chlorpromazine-equivalent doses (1053.9 vs. 1377.2 mg/day; p = 0.013) were significantly lower. Medication-related QoL improved significantly in four PROMPT-QoL domains (p = 0.015–0.041). |
| Stuhec, 2025 | Medication review (type 3) | Total medications decreased by 6.6% (p < 0.001), X-type DDIs by 75.8%, D-type DDIs by 56.9%, PIMs by 29.5% (PRISCUS) and 17.5% (Beers). Pharmacists made a mean of 5.6 recommendations/patient, mainly drug discontinuation (49%). Adherence to treatment guidelines for somatic comorbidities improved from 3.3–13.2% to 50.0–72.6%. The acceptance rate was 59.2%. |
| Hahn, 2018 | Participation in multidisciplinary ward rounds | 101 pharmaceutical interventions were performed with 100% acceptance. Moderate DDIs decreased by 44% and PRISCUS medications by 42% during hospitalization, although changes were not statistically significant after discharge. |
| Stuhec, 2021 | Participation in multidisciplinary ward rounds | DRPs decreased from 315 to 20 (−93.8%). Recommendation acceptance rates were 91.2% (effectiveness), 97.2% (inappropriate treatment), and 100% (safety), with an overall acceptance of 93.7%. 70.5% of recommendations remained implemented after 3 months, with no intervention-related adverse events. |
| Stuhec, 2024 | Participation in multidisciplinary ward rounds | 280 pharmacist recommendations were made (1.5 recommendations/patient); 86.4% of expressed DRPs were resolved. Recommendation acceptance was 88.9% at discharge and 63.2% after 3 months. Adherence to treatment guidelines for somatic comorbidities increased significantly (p < 0.05). |
| **First Author, Year** | **Type of intervention** | **Outcomes** |
| Lizer, 2007 | Medication reconciliation | Pharmacists identified significantly more medications than nurses at admission (5.3±3.7 vs. 4.0±3.2; p < 0.05). 158 medication discrepancies were identified (mean 2.9±2.5/patient), most commonly omitted medications (48%) and incorrect doses (31%). |
| O'Connell, 2021 | Medication reconciliation | Medication reconciliation in psychiatric patients, performed either before admission or within 24 hours of admission, was not associated with a statistically significant reduction in inpatient length of stay, although in one of the prospective periods (PP2) the average length of hospitalization decreased by 1.52 days. In contrast, pharmacist-led medication reconciliation significantly reduced the number of medication errors (by 261% in PP1 and by 348% in PP2) as well as the number of discharge discrepancies (by 934% in PP1 and by 968% in PP2) compared with the retrospective control group (RP), with all differences being statistically significant (p < 0.001). |
| Oliveira, 2022 | Medication reconciliation | Following medication reconciliation, a total of 560 clinically sound intentional discrepancies were identified, along with 359 discrepancies requiring further clarification from prescribers. Among these, drug omissions were the most frequent (302; 84.12%), followed by drug substitutions (20; 5.57%), dose changes (25; 6.96%), and changes in dosing frequency (12; 3.34%). |
| Vargas, 2023 | Medication reconciliation | 82 medication errors were identified; 90.2% had the potential to cause harm if uncorrected. Dosing errors (32.9%) and medication omissions (25.6%) were the most frequent. 71.9% of errors had potential post-discharge consequences. |
| Saklad, 1984 | Clinical pharmacy intervention | Outcomes were assessed at day 1 (admission), day 14, and day 30 by comparing the pre-intervention group (1978, before implementation of clinical pharmacy services) with the post-intervention group (1979, after implementation). On day 1, the number of antipsychotics per patient decreased from 1.26 ± 0.63 to 0.97 ± 0.41 (−22.5%), and anticholinergic use from 0.79 ± 0.63 to 0.37 ± 0.49 (−53.2%). By day 14, the total number of drugs (3.60 ± 0.91 to 2.57 ± 1.20; −28.6%), daily doses (8.12 ± 3.51 to 4.86 ± 1.96; −40.1%), and antipsychotics (1.69 ± 0.68 to 1.14 ± 0.48; −32.5%) decreased. By day 30, total drugs (3.89 ± 0.87 to 2.57 ± 1.15; −33.9%), daily doses (8.42 ± 3.42 to 4.85 ± 2.54; −42.4%), antipsychotics (1.84 ± 0.60 to 1.14 ± 0.53; −38.0%), and anticholinergic use (0.68 ± 0.58 to 0.46 ± 0.66; −32.4%) were reduced. Length of hospital stay decreased from 77.2 ± 93.8 to 58.6 ± 71.6 days (−23.6%; p < 0.10), while 1-year readmission decreased significantly from 0.68 to 0.30 per patient (p < 0.05). |
| Hahn, 2013 | DDIs review | Following the clinical pharmacist intervention, the number of clinically relevant DDIs decreased by 78% (from 263 to 58; p < 0.001), while the number of potential DDIs decreased from 439 to 194 (p < 0.001). |

**Abbreviations:** ADEs, adverse drug events; ADRs, adverse drug reactions; ARV, antiretroviral; DDIs, drug–drug interactions; DRPs, drug-related problems; HIV, human immunodeficiency virus; OI, opportunistic infection; PIMs, potentially inappropriate medications; PROMT-QoL, Patient-Reported Outcomes Measure of Pharmaceutical Therapy for Quality of Life; QoL, quality of life.

Table. Key outcomes of the included studies.
