# Supplementary material for: Impact of clinical pharmacist interventions in psychiatric hospitals on clinical and medication-related outcomes: a systematic review and meta-analysis
Source: Front Pharmacol. 2026 Oct 8;17:1945082. doi: 10.3389/fphar.2026.1945082 (PMC13687401; doi:10.3389/fphar.2026.1945082)
Supplement: Supplementary file 2 [file Supplementaryfile2.docx]

Complete Search Strategies and Additional Data.

Fig. S1. Funnel plot for the meta-analysis of the mean difference in the number of medications.

Fig. S2. Funnel plot for the meta-analysis of DRP reduction percentage.

Fig. S3: Leave-one-out sensitivity analysis of the percentage of DRPs reduced following clinical pharmacist intervention.

A leave-one-out sensitivity analysis was performed to assess the influence of individual studies on the pooled estimate of DRP reduction (Figure 3). Sequential omission of each study resulted in pooled DRP reductions ranging from **64.2% to 73.4%**. The exclusion of **Stuhec, 2021** had the greatest impact on the pooled estimate and reduced between-study heterogeneity from **96.5% to 94.3%**, although heterogeneity remained very high. Omitting any other study produced only modest changes in the pooled estimate and heterogeneity (I² ranging from 96.3% to 97.1%). Overall, the findings indicate that the observed effect was robust and was not driven by any single study, despite the substantial between-study heterogeneity.
